# Analysis of the efficacy and risk factors of surgical treatment of recurrent UPJO in adults

**DOI:** 10.1007/s11255-022-03439-3

**Published:** 2022-12-26

**Authors:** Wenzhi Gao, Lei Zhang, Yuhui He, Tai Tian, Zhihua Li, Liangliang Bai, Ying Shen, Chen Huang, Bing Wang, Peng Zhang, Ninghan Feng, Xuechao Li, Yuexian Guo, Xuesong Li

**Affiliations:** 1 Department of Urology, Peking University First Hospital, Institute of Urology, Peking University, National Urological Cancer Center, No.8 Xishiku Street, Xicheng District, Beijing, 100034 China; 2 Department of Urology, The Third Hospital of Hebei Medical University, Ziqiang Road, Qiaoxi District, Shijiazhuang City, 050000 Hebei Province China; 3 Department of Urology, Jian Gong Hospital, Beijing, 100034 China; 4 Department of Urology, Peking University First Hospital, Miyun Campus, Beijing, 100034 China; 5 Department of Urology, Emergency General Hospital, Beijing, 100034 China; 6 Wuxi No. 2 People’s Hospital of Nanjing Medical University, Nanjing Medical University, Jiangsu, 214002 China; 7 Department of Urology, The Fifth Medical Centre of Chinese PLA General Hospital, Beijing, 100034 China

**Keywords:** Uretero-pelvic junction obstruction, Secondary surgery, Risk factor analysis, Pyeloplasty, Balloon dilation

## Abstract

**Background:**

To compare the efficacy of secondary pyeloplasty and balloon dilation and to analyze the risk factors for secondary surgical failure in patients with recurrent uretero-pelvic junction obstruction (UPJO).

**Methods:**

We retrospectively analyzed 65 patients with recurrent UPJO who underwent secondary surgery between September 2011 and March 2019, of whom 33 had complete baseline data and follow-up data. General clinical information, perioperative data, and follow-up results were collected from patients. Risk factors for surgical failure in patients with recurrent UPJO were analyzed using logistic regression.

**Results:**

The failure rates of secondary pyeloplasty and balloon dilation in secondary surgery were 16.7% and 33.3%, respectively. Univariate analysis showed that ureteral stenosis length and operative time were associated with secondary pyeloplasty and balloon dilatation failure (*p* < 0.05), and ureteral stenosis length was an independent risk factor for secondary pyeloplasty failure (OR = 0.074, 95% CI: 0.006–0.864, *p* = 0.038). In the balloon dilation group, treatment failure rates were significantly lower in patients with stenotic segment lengths less than 1 ± 0.32 cm than in patients with stenotic segment lengths greater than 1 ± 0.32 cm (*p* = 0.019).

**Conclusions:**

The secondary pyeloplasty may provide better benefit. Ureteral stricture length is an independent risk factor for failure of secondary pyeloplasty and a potential risk factor for balloon dilatation. Operation time is a potential risk factor for pyeloplasty and balloon dilatation.

## Introduction

Uretero-pelvic junction obstruction (UPJO) can lead to accumulation of urine in the renal pelvis and reduced flow of urine from the renal pelvis into the ureter. Long-term urinary tract obstruction causes hydronephrosis, chronic infection, urolithiasis, reduced kidney function, and lower back pain, which may affect the quality of life of patients [1]. The most common treatment for UPJO is pyeloplasty after the onset of symptoms or the discovery of severe hydronephrosis that affects renal function [2–4], but pyeloplasty has a failure rate of 2.5–10% [5–7]. The recurrence of symptoms, such as low back pain and hydronephrosis in recurrent UPJO, seriously affects the quality of life of patients [6]. Although the number of patients with recurrent UPJO is small but the treatment is difficult and the patients' needs are urgent, it is necessary to analyze the risk factors for surgical treatment failure of patients with recurrent UPJO.

Several treatment approaches exist for patients with recurrent UPJO include secondary pyeloplasty, retrograde cavernous pyelotomy, pyelocystic anastomosis, balloon dilation, percutaneous nephrostomy, and nephrectomy [5]. Among these, pyeloplasty has a higher success rate and fewer complications than other treatments, and balloon dilation has the advantage of less invasion and more acceptable to the patient, both of which are more common in patients with recurrent UPJO. The failure rate of secondary surgery in patients with recurrent UPJO ranges from 0% to 22.2% [8–10], and factors contributing to recurrent UPJO failure may be related to renal stones, preoperative renal insufficiency, diabetes mellitus, intraoperative bleeding, and length of ureteral stenosis.

There is no definitive relation between ureteral stricture length and surgical failure in the previous studies. In this study, the prognosis for patients with a stenosis of 1.5 cm or less was found to be very favorable, but the options of treatment for patients with 2–3 cm need to be further explored. There are no clear guidelines on the effect of ureteral stricture length on the failure rate of balloon dilatation, and this study further investigates the effect of different ureteral stricture lengths. This study further explores the risk factors for surgical failure in the treatment of pyeloplasty and balloon dilatation, and provides some reference for clinicians to develop treatment plans.

## Methods

### Study population

Sixty-five consecutive patients with recurrent UPJO underwent secondary surgical treatment after a failed first pyeloplasty at the Department of Urology, Peking University First Hospital, Emergency General Hospital, Fifth Medical Centre of Chinese PLA General Hospital and Jian Gong Hospital between September 2011 and March 2019, of whom 33 patients had complete baseline and follow-up data. Clinical data, including demographics, procedure details, perioperative records, complications, and patient outcomes, were collected in our Reconstruction of the Urinary Tract: Epidemiology and Result (RECUTTER) database [11]. The quality of life and hydronephrosis in patients with recurrent UPJO secondary surgery were collected and patients were divided into two groups (failure group and non-failure group). The failure group was defined as those who developed worsening hydronephrosis and symptoms of flank pain or those who had a nephrostomy and underwent a third repair surgery for symptoms [12–16]. Patients who could not meet these above-mentioned criteria were classified in non- failure group. Inclusion criteria were as follows: 1. Patients who developed ipsilateral upper urinary tract obstruction after surgical treatment of UPJO and were proposed for reoperation; 2. Patients aged over 18 years; 3. Patients who had been operated on for more than 3 years. The exclusion criteria included: 1. any contraindication against surgery; 2. combined pregnancy; 3. incomplete data. The final data were considered valid for 33 patients, including 24 patients who underwent pyeloplasty and nine patients who underwent balloon dilation.

Information regarding patients’ clinical status at the latest clinical follow-up available was collected by telephone interviews and the true validity of the clinical data was verified in 33 patients. Preoperative clinical variables in this study included gender, age, BMI, diabetes, coexisting stones, primary surgical approach, degree of hydronephrosis (NA, mild/moderate, severe), preoperative renal function (preoperative creatinine, preoperative urea nitrogen, preoperative blood eGFR), duration of surgery, bleeding, and length of ureteral stricture. To explore the risk factors for surgical failure in patients with recurrent UPJO, using patients' surgical failure and non-failure as the final outcome.

During pyeloplasty, the pelvic ureteral junction is found above the posterior gonadal vein, the periureteral scar is separated, and the length of the ureteral stenosis is measured, the lower corner of the pelvis is cut 2–4 cm, the ureter is cut 1.5 cm transversely below the stenotic junction, and then, the ureter is cut longitudinally for approximately 2 cm, the lower corner of the pelvis and the lowermost part of the ureter cut longitudinally are closed with the first suture, then the posterior wall is anastomosed, and a D-J tube is placed after. The anterior wall is then anastomosed and the obstructed area is finally cut out with a circular suture. The balloon was dilated with a 24F diameter balloon at 25 atmospheric pressure for 3 min and the stenosis was dilated in a retrograde path, after which two 6F/26 cm D-J tubes were left in place.

This study was conducted in accordance with the principles of the Declaration of Helsinki (revised 2013) and was approved by the Ethics Committee of Peking University First Hospital, and individual consent for retrospective analysis of this study was waived.

### Statistical analysis

Excel software (version 2019) was used for data management and SPSS software (version 22) was used for analyzing data. Quantitative variables included age, BMI, preoperative renal function (preoperative creatinine, preoperative urea nitrogen, preoperative blood eGFR), duration of surgery, bleeding, and length of ureteral stricture, and qualitative variables included gender, diabetes mellitus, degree of hydronephrosis, combined urinary stones, and mode of first surgery. Normally distributed data are expressed as mean ± standard deviation; skewed data are described as median and interquartile range. For continuous variables, variables with normal distributions were analyzed using the *t* test and those with abnormal distributions were analyzed using the Mann–Whitney *U* test. Categorical variables were analyzed by Fisher’s exact probability test. Binary logistic regression analysis (*p* < 0.05) and multivariable logistic regression analysis (*p* < 0.1) were used for independent risk factor analysis.

## Results

### Comparison of the efficacy of pyeloplasty and balloon dilation

The clinical data of the 33 patients with recurrent UPJO are shown in Table 1. The failure rate for secondary pyeloplasty was 16.7% and for secondary balloon dilatation 33.3%. Preoperative creatinine, preoperative urea nitrogen, and preoperative eGFR were used together to reflect preoperative renal function, which was not significantly different in the pyeloplasty and balloon dilatation groups (*p* > 0.05). There was also no significant difference in the degree of hydronephrosis between the pyeloplasty and balloon dilatation groups (*p* > 0.05).

**Table 1:** Demographic and clinical characteristics of 33 patients

Variable	Pyeloplasty	Balloon dilation	95%CI	*p* value
Patients, *n* (%)	24 (73)	9 (27)		
Mean age (years)	30.92 ± 8.12	25.22 ± 5.14	− 14.656	0.062
BMI (kg/m^2^)	23.49 ± 2.99	23.64 ± 3.01	− 4.771	0.900
Gender, *n* (%)				0.407
Male	15 (62.5)	7 (77.8)		
Female	9 (37.5)	2 (22.8)		
Degree of hydronephrosis				0.245
NA	4 (16.7)	3 (33.3)		
Mild/moderate	5 (20.8)	3 (33.3)		
Severe	15 (62.5)	3 (33.3)		
Preoperative creatinine (μmol/L)	80.86 ± 17.63	73.96 ± 12.01	− 20.568	0.289
Preoperative bun (mmol/L)	5.19 ± 1.09	4.32 ± 1.30	− 1.833	0.062
Preoperative eGFR (mL/min)	101.36 ± 18.85	113.78 ± 12.24	− 27.724	0.077
Surgery time (min)	180.33 ± 60.49	40.44 ± 17,393	97.772 − 182.056	< 0.001
Intraoperative blood loss (mL)	27.36 ± 33.53	4.44 ± 5.27	− 46.626	0.052
Length of ureteral stenosis (cm)	1.38 ± 0.70	1.66 ± 1.27	− 1.263	0.569
Surgical outcome, *n* (%)				0.312
Non-failure rate	20 (83.3)	6 (66.7)		
Failure rate	4 (16.7)	3 (33.3)		

*eGFR* estimated glomerular filtration rate; *SCr* serum creatinine; *BUN* blood urea nitrogen; *BMI* body mass index; *NA* not available

### Analysis of risk factors for failure of pyeloplasty

The clinical data of the 24 patients with recurrent UPJO who underwent secondary pyeloplasty are shown in Table 2. The length of ureteral stenosis was 2.25 ± 0.86 cm in the failed group and 1.20 ± 0.53 cm in the non-failed group, with a significant difference between the two groups (*p* < 0.05). A univariate logistic regression analysis showed that ureteral stenosis length was an independent risk factor for second pyeloplasty failure (OR = 0.074, *p* = 0.038), as shown in Table 3. The operative time was 244 ± 53.48 min in the failed group and 167.60 ± 54.34 min in the non-failed group, with a significant difference between the two group (*p* < 0.05), and the patient’s operative time could be a potential influencing factor for second pyeloplasty failure.

**Table 2:** Demographic and clinical characteristics of 24 patients

Variable	Non-failure group	Failure group	*p* value
Patients, *n* (%)	20 (83.33)	4 (16.67)	
Mean age (years)	29 (26, 37.25)	29 (24.5, 32.75)	0.573
BMI (kg/m^2^)	23.25 (20.93, 25.53)	22.95 (20.43, 26.98)	0.978
Gender, *n* (%)			0.572
Male	12 (60)	3 (75)	
Female	8 (40)	1 (25)	
Diabetes, *n* (%)			0.186
Yes	1 (5)	1 (25)	
No	19 (95)	3 (75)	
Urinary stones, *n* (%)			0.624
Yes	3 (17.65)	1 (25)	
No	17 (82.35)	3 (75)	
The first surgical procedure, *n* (%)			0.624
Open	3 (17.65)	1 (25)	
Laparoscopic/robot assist	17 (82.35)	3 (75)	
Degree of hydronephrosis			0.718
NA	1 (25)	3 (15)	
Mild/moderate	1 (25)	4 (20)	
Severe	2 (50)	13 (65)	
Preoperative SCr (μmol/L)	85.83 ± 17.58	79.87 ± 17.93	0.549
Preoperative BUN (mmol/L)	6.14 ± 1.08	5.01 ± 1.02	0.056
Preoperative eGFR (mL/min)	90.02 ± 22.09	103.62 ± 17.91	0.194
Surgery time (min)	166 (134.25, 195)	239 (196.75, 296.25)	0.017
Intraoperative blood loss (mL)	12.5 (10, 20)	16.5 (10, 43.25)	0.795
Length of ureteral stenosis (cm)	2.25 ± 0.87	1.20 ± 0.53	0.004

**Table 3:** Univariate and multivariate logistic regression analysis of risk factors leading to surgical failure in secondary pyeloplasty

Variable	Univariate			Multivariate		
	OR	95% CI	*p* value	OR	95% CI	*p* value
Mean age	1.047	0.898–1.221	0.558			
BMI	1.006	0.696–1.452	0.977			
Gender (male/female)	0.500	0.440–5.700	0.577			
Diabetes (no/yes)	6.333	0.307–130.751	0.232			
Urinary stones (no/yes)	1.889	0.144–24.793	0.628			
The first surgical procedure (no/yes)	1.889	0.144–24.793	0.628			
Preoperative SCr	0.982	0.938–1.040	0.535			
Preoperative BUN	0.245	0.051–1.181	0.08	0.036	0.000–4.082	0.168
Preoperative eGFR	1.039	0.980–1.102	0.199			
Surgery time	0.986	0.970–1.003	0.107			
Intraoperative blood loss	1.005	0.967–10.45	0.786			
Length of ureteral stenosis	0.074	0.006–0.864	0.038	0.004	0.000–5.311	0.133

Preoperative creatinine, preoperative urea nitrogen, and preoperative eGFR were used together to reflect preoperative renal function, which was not significantly different between the failed and non-failed groups (*p* > 0.05). There was also no significant difference in the degree of hydronephrosis between the failed and non-failed groups (*p* > 0.05).

### Analysis of risk factors for failure of balloon dilation

The clinical data of nine patients with recurrent UPJO undergoing balloon dilation are shown in Table 4. The operative time of balloon dilation was 60 ± 19.5 min in the failed group and 31 ± 5.46 min in the non-failed group, with a significant difference between the two groups (*p* = 0.006). The length of ureteral stricture was 2.7 ± 1.04 cm in patients with failed balloon dilation and 1 ± 0.32 cm in the non-failed group, with a significant difference between the two groups (*p* = 0.019).

**Table 4:** Demographic and clinical characteristics of nine patients

Variable	Patient 1	Patient 2	Patient 3	Patient 4	Patient 5	Patient 6	Patient 7	Patient 8	Patient 9
Age	33	22	28	30	23	30	23	19	19
BMI (kg/m^2^)	26	26.7	20.6	23.24	22.9	19.7	24.5	20.7	28.4
Gender	Female	Male	Male	Male	Male	Female	Male	Male	Male
First surgical procedure	Open	Open	LA/Robot	LA/Robot	Open	LA/Robot	Open	LA/Robot	LA/Robot
Degree of hydronephrosis	Severe	Moderate	Moderate	NA	Severe	NA	NA	Severe	Moderate
Preoperative creatinine (μmol/L)	71	72.9	82.8	80	62	50	86.9	74	86
Preoperative bun (mmol/L)	2.63	4.57	3.6	3.79	3.9	3.9	4.33	4.83	7.37
Preoperative eGFR (mL/min)	97	101	108	114	133	125	107	127	112
Surgery time (min)	60	40	79	30	30	40	32	30	23
Length of ureteral stenosis (cm)	3	1.5	3.5	1.5	0.5	1	1	1	1
Surgical outcomes	Failure	Failure	Failure	Non-failure	Non-failure	Non-failure	Non-failure	Non-failure	Non-failure

The preoperative blood eGFR was 75.6 ± 6.33 μmol/L in the failed group and 73.15 ± 14.57 μmol/L in the non-failed group; the preoperative urea nitrogen was 3.6 ± 0.97 mmol/L in the failed group and 4.69 ± 1.37 mmol/L in the non-failed group, with no significant difference between the groups (*p* > 0.05); the preoperative blood eGFR was 102 ± 5.57 mL/min in the failed group and 120 ± 10.11 mL/min in the non-failed group, with no significant difference between the groups (*p* > 0.05). There was also no significant difference in the degree of hydronephrosis between the failed and non-failed groups (*p* > 0.05).

## Discussion

Obstruction of the renal pelvic junction can block the drainage of urine produced by the kidneys, which in turn increases the pressure in the renal pelvis, causing damage to kidney function and affecting the patient's quality of life with symptoms of low back pain. Adult patients with UPJO are mainly found to have UPJO through physical examination and after symptoms such as low back pain are present, and are often treated with pyeloplasty after detection. However, there is a 2.5–10% failure rate of pyeloplasty, and the renal function and quality of life of patients who fail do not improve and can only improve renal function and quality of life through secondary surgery. This article examines the risk factors that influence secondary surgery and provides an important basis for clinicians to make treatment plans.

In this study, when comparing the outcomes after secondary pyeloplasty and balloon dilation, the failure rates were higher for balloon dilation, with no significant difference in patient baseline data. Due to sample size limitations, there was no statistically significant difference in failure rates between the two procedures (*p* > 0.05), but in the context of clinical practice and failure rates, pyeloplasty provided better benefit than balloon dilation.

The effect of the length of the ureteral stenosis on the success rate of secondary pyeloplasty has not been clearly reported in the past. In the present study, it was found that all procedures were successful for ureteral stenoses of 1.5 cm or less, while the failure rates were 22.22% and 40% for ureteral stenoses of 1.51–2 cm and 2.01–3 cm, respectively. The benefits of pyeloplasty in patients with ureteral stenosis of 1.5 cm or less are significant, while those with ureteral stenosis of 1.51–3 cm need to be further investigated. In previous studies, the effective rate of balloon dilatation was 42% for ureteral stenoses > 1.5 cm and only 25% for stenoses > 2 cm [17, 18], compared with a 0% failure rate for balloon dilatation of ureteral stenoses 0–1 cm, 50% for 1.1–1.5 cm and 100% for 1.51–3 cm in this study. We considered that balloon dilation gives good benefit when the length of ureteral stricture is < 1 cm and that patients with > 1 cm have poorer long-term post-operative results. Balloon dilation may be the best procedure for short-term benefit in patients with ureteral strictures up to 1 cm in length, but long-term results need to be further investigated at follow-up.

All ureteral repair procedures are surgically difficult [19], with recurrent UPJO being among the most difficult to manage in ureteral repair procedures. Treatment options for complex recurrent UPJO also include Lingual Mucosal Graft Ureteroplasty and ileal ureter replacement, which are both more time-consuming and difficult to treat [20, 21]. Ahmed Hammady et al. [22] found that when comparing patients with primary UPJO to those with recurrent UPJO, the severity of peripelvic ureteral fibrosis in patients with recurrent UPJO led to increased operative difficulty, significantly longer operative times (*p* = 0.01), and higher complications (*p* = 0.02) than in patients with primary UPJO. The varying degree of peripelvic fibrosis affects to some extent the difficulty of secondary pyeloplasty and therefore the operative time. The difference in operative time between the failed and non-failed pyeloplasty groups in this study (*p* < 0.05) may be related to the degree of peripelvic fibrosis after the primary surgery, Therefore patients with severe peripelvic ureteral scarring deciding to undergo secondary surgery should be considered carefully, and adequate communication should be made with patients before surgery to inform them of the possibility of surgical failure and to avoid post-operative doctor–patient conflicts. The difference in operative time between the failed and non-failed groups in balloon dilatation (*p* < 0.05) may be related to the longer operative time leading to increased ureteral ischemia; therefore, the operative time should be controlled to complete the surgery as soon as possible when balloon dilatation is performed.

In patients with primary UPJO, factors, such as renal function, age, and concomitant stones, may have an impact on the outcome of the procedure. However, post-operative recurrence in patients with UPJO is influenced by a number of factors, and there are some differences in baseline data in patients with recurrent UPJO compared to patients with primary UPJO. Therefore, the risk factors for surgical failure may differ between patients with primary UPJO and those with recurrent UPJO. Ortapamuk et al. [23] found better recovery of renal function after preoperative fractional renal function greater than 30% pyeloplasty; Li et al. [24] found that among 138 adult UPJO patients older than 35 years of age had poorer recovery of renal function postoperatively than those younger than 35 years; Chow et al. [1] showed that potential risk factors for patients undergoing first-time UPJO pyeloplasty included preoperative renal insufficiency, patient age, early leakage, concomitant renal calculi, and abnormal renal histology. The above studies were all conducted on patients with primary UPJO. In this study, age, gender, degree of hydronephrosis, and BMI were not found to have a significant effect on the outcome of the procedure during secondary pyeloplasty and balloon dilation, suggesting that older and heavier patients should not be excluded from pyeloplasty and balloon dilation. As the degree of hydronephrosis was relatively severe in both patients who underwent failed and non-failed secondary surgery, this may be the reason why the degree of hydronephrosis is not a risk factor for failure of pyeloplasty and balloon dilatation.

There are still some limitations in this study. In this retrospective study, the failure and non-failure groups were set up based on ultrasound-indicated changes in hydronephrosis and the patient's symptoms, and could not be grouped more precisely according to renal function; although the number of patients undergoing secondary surgery after UPJO recurrence is rare, secondary surgery is difficult and has a significant impact on patients' quality of life, so a prospective cohort to expand the sample size is particularly important. A prospective cohort of patients undergoing secondary repair has been established to expand the sample size while rigorously collecting creatinine and diuretic renal dynamics to better define the quality of life and renal function status of patients after surgery.

## Conclusion

The benefits of pyeloplasty are likely to be better than those of pyeloplasty and balloon dilation. The present evidence suggests that ureteral stenosis length is an independent risk factor for failure of secondary pyeloplasty and that operation time may be a potential risk factor. Ureteral stenosis length and operation time may be potential risk factors for failure of secondary balloon dilatation. The length of ureteral stenosis and operation time have an impact on the outcome of pyeloplasty and balloon dilatation, so preoperative assessment of the patient's ureteral stenosis length by cis-retrograde imaging and assessment of peripelvic ureteral fibrosis by Magnetic Resonance Imaging may be helpful in clinical practice.

## Data Availability

Original data are available by request to corresponding author.
